# Mistakes in translation: Reflections on mechanism

**DOI:** 10.1371/journal.pone.0180566

**Published:** 2017-06-29

**Authors:** Yizhou Liu, Joshua S. Sharp, Duc H-T. Do, Richard A. Kahn, Harald Schwalbe, Florian Buhr, James H. Prestegard

**Affiliations:** 1 Complex Carbohydrate Research Center, University of Georgia, Athens, Georgia, United States of America; 2 Department of BioMolecular Sciences, University of Mississippi, Oxford, Mississippi, United States of America; 3 Department of Food Science and Technology, University of Georgia, Athens, Georgia, United States of America; 4 Department of Biochemistry, Emory University School of Medicine, Atlanta, Georgia, United States of America; 5 Institute for Organic Chemistry and Chemical Biology, Johann Wolfgang Goethe-University, Frankfurt, Germany; University of Lethbridge, CANADA

## Abstract

Mistakes in translation of messenger RNA into protein are clearly a detriment to the recombinant production of pure proteins for biophysical study or the biopharmaceutical market. However, they may also provide insight into mechanistic details of the translation process. Mistakes often involve the substitution of an amino acid having an abundant codon for one having a rare codon, differing by substitution of a G base by an A base, as in the case of substitution of a lysine (AAA) for arginine (AGA). In these cases one expects the substitution frequency to depend on the relative abundances of the respective tRNAs, and thus, one might expect frequencies to be similar for all sites having the same rare codon. Here we demonstrate that, for the ADP-ribosylation factor from yeast expressed in *E*. *coli*, lysine for arginine substitutions frequencies are not the same at the 9 sites containing a rare arginine codon; mis-incorporation frequencies instead vary from less than 1 to 16%. We suggest that the context in which the codons occur (clustering of rare sites) may be responsible for the variation. The method employed to determine the frequency of mis-incorporation involves a novel mass spectrometric analysis of the products from the parallel expression of wild type and codon-optimized genes in ^15^N and ^14^N enriched media, respectively. The high sensitivity and low material requirements of the method make this a promising technology for the collection of data relevant to other mis-incorporations. The additional data could be of value in refining models for the ribosomal translation elongation process.

## Introduction

It is well recognized that translation of mRNAs to the polypeptides of functioning proteins is a carefully controlled process with promoters binding to sites upstream of coding sequences, the recruitment of numerous effector proteins to the ribosomal surface and post-translational modification of some of these proteins [1, 2]. Less appreciated is the control that may be exerted by the use of different codons for a given amino acid and variations in the availability of tRNAs that bind to these codons. We encountered a consequence of this variation in the course of expressing a eukaryotic protein in a bacterial host for NMR structural studies, namely that, in the absence of adequate supplies of complementary tRNAs, mistakes in translation are made; in our case, addition of a lysine at sites where an arginine belongs. This phenomenon has been observed previously, as it leads to extra crosspeaks in the ^1^H-^15^N 2D NMR spectra commonly used as a structural fingerprint of the protein studied [3], and sometimes to incorrect attribution of these peaks to alternate conformational forms. What is striking in our observation is that the frequency of mistakes at different positions in the coding sequence varies, even when the rare codons for the arginine to be added are the same. This suggests additional sequence dependent control of translation and the possibility that examination of the frequency of mistakes could shed light on control mechanisms. We report here data on the frequency of mistakes and examine possible mechanistic explanations.

Biological organisms utilize all 64 triplet combinations of the common 4 DNA nucleotides to code for 20 amino acids plus the stop signal. This unavoidably leads to degeneracy of genetic coding in the sense that multiple triplets code for the same amino acid. However, the usage of these degenerate (synonymous) codons is biased, even within a single organism. Those used less often are referred to as “rare codons”. The incorporation of rare codons into the mRNA for a particular protein can potentially serve a number of purposes [4]. A correlation of rare codon usage with protein secondary structure in higher organisms was identified a number of years ago [5, 6], and this raised the possibility that codon usage is related to protein folding. The prevailing explanation is that the availability of the complementary tRNA affects the rate of translation, which could be coupled to co-translational folding and eventually protein function [7, 8]. Indeed, silent mutations (or synonymous substitutions), which lead to the introduction of codons for more or less abundant tRNAs have been linked to altered protein activities and human diseases [8–10].

In micro-organisms, the codons used tend to be evolutionarily optimized to utilize the more abundant tRNA species and there is significant codon bias [11]. The bias in metazoans is, however, quite different and eukaryotic proteins frequently contain codons rarely used by bacteria. When eukaryotic proteins are expressed in *E*. *coli*, an enhanced level of mis-translation can occur. The most frequently observed case is arginine to lysine substitution, where the arginine rare codon AGA is erroneously recognized by ${\mathrm{tRNA}}_{UUU}^{Lys}$ [12–14]. Mis-incorporation of glutamine (CAG) for arginine (CGG) has also been reported [15, 16], and the impact in areas such as biopharmaceutical production has been discussed [17]. A simple explanation for this phenomenon is that the lack of arginine tRNA's for these codons allows other more abundant amino-acyl-tRNA complexes (EF-Tu:GTP:tRNA) to out compete for the ribosomal A site and accomodate a near-cognate tRNA. Competition at an early point in the docking of a new tRNA complex is supported by the observation that these mis-translations are effectively suppressed in *E*.*coli* strains supplemented with genes coding for rare codon tRNAs [13, 15].

If simple competition for a rare codon site were the end of the story, the level of mistakes would be the same for each occurrence of a rare codon. Recently, significant effort has gone into the development of kinetic and statistical models for the translational process [18–20]. While these models focus on simulating translation elongation rates, and make comparisons only to data on net elongation rates, extension to the prediction of different mis-incorporation levels at different instances of the same rare codon would seem possible. The models incorporate as many as 11 discrete steps in the translation elongation process. Some of these clearly can affect fidelity in translation. For example, EF-Tu:GTP:tRNA ternary complexes initially compete non-specifically for binding to the ribosomal decoding site, making the relative concentration of cognate versus non-cognate or near-cognate complexes a factor in the elongation rate. Because these concentrations can be considered local concentrations, clustering of rare sites in the coding sequence could deplete cognate complexes, increasing the probability of a near- or non-cognate complex occupying the decoding site, and this probability could be reflected in the frequency of miss-incorporation. Some supporting evidence for the effect of local depletion exists in the observation that clustering of identical rare codons increases the probability of a frame-shift during translation [21].

Selection of the proper cognate complex is known to depend not only on the energetics of base-pair formation, but on structural shifts in the decoding site that favor the proper complex [22, 23]. Subsequent steps, which include activation of EF-Tu for GTP hydrolysis, accommodation of the tRNA in the A site of 50S ribosomal subunit, peptide bond formation, and movement of the tRNA-mRNA pairs to subsequent tRNA binding sites, could also contribute directly to fidelity of decoding. Particularly at the GTP hydrolysis step forward movements of cognate complexes are known to occur at higher rates than near-cognate complexes, allowing more time for release of a near-cognate tRNA-EF-Tu-GTP complex and replacement with the proper cognate complex [24–26]. The contribution of such selective steps to fidelity could be diminished by processes that stall progression in a sequence dependent manner and make differences in rates less relevant. For example, stalling by the presence of other ribosomes on the same mRNA (polysomes) or the necessary un-wrapping of mRNA secondary structures, could eliminate any advantage of moving cognate complexes forward more rapidly. Also, the particular amino acid in the P site, C-terminal to the peptide being generated, can also affect the rate of peptide bond formation [27], and possibly the frequency of miss-incorporation. Mechanisms by which rates of these additional steps are affected, and particularly the effects of upstream and downstream sequences are not fully understood. However, some progress has been made in understanding the effects of mutations quite distant from the EF-Tu binding site that accelerate GTP hydrolysis, particularly for near-cognate complexes [28]. These effects are believed to be transmitted by subtle shifts of ribosomal structural elements. The ribosome also contacts a significant stretch of mRNA [29], as well as nascent peptides during synthesis [30], and it would be possible that the effects of these contacts could be similarly transmitted to elements responsible for maintaining fidelity in translation.

**Fig 1:**
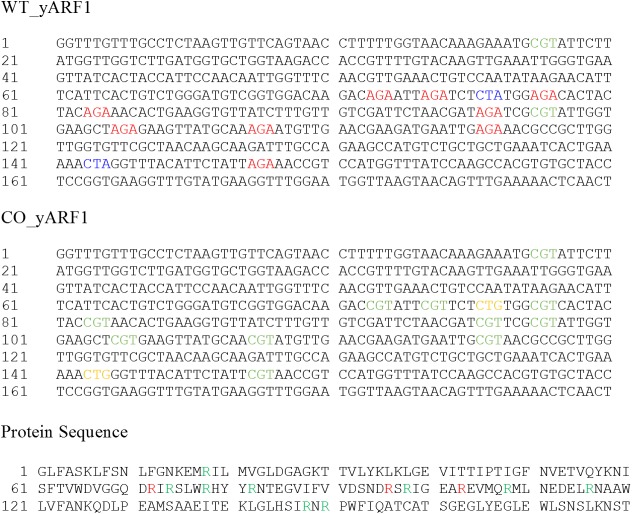
DNA sequences for wild type (WT_yARF1) and codon optimized (CO_yARF1) yeast ARF1. Numbering is for the corresponding amino acids beginning after the initial methionine. The protein sequence of the wild type protein is provided to facilitate translation. Red denotes an arginine codon that is rare in *E*. *coli*. Blue denotes a leucine codon that is rare in *E*. *coli*. Green denotes the more *E*. *coli* abundant arginine codon substituted in CO_yARF1. It is rare in yeast, but it also exists in two sites for WT_yARF1. Yellow denotes the more *E*. *coli* abundant leucine codon substituted in CO_yARF1.

The data which we offer as a potential means of evaluating models and identifying contributors to translational errors involves a quantitative analysis of the arginine-to-lysine mis-incorporation rates in the bacterially expressed yeast (*Saccharomyces cerevisiae*) ADP-ribosylation factor (yARF1), a protein that contains 9 arginines coded by AGA (see Fig 1). The data were acquired using a novel parallel expression procedure in which the native gene containing rare codons was expressed in an *E*. *coli* BL21 cell line grown on a ^15^N supplemented medium. In parallel, a codon optimized gene that included AGA codons being substituted with common CGT codons, was expressed in the same BL21 cell line, but grown on a natural abundance (^14^N) medium. yARF1 products were isolated and mixed for MS analysis of isotope ratios in arginine containing peptides coming from various sequences having rare and abundant codons in the native sequence. Interestingly, arginine is replaced with another amino acid (lysine) in the 9 sites at different frequencies. Because this substitution frequency is potentially correlated with site-specific translation rates, it may provide insight into translation control and the time course of co-translational peptide folding.

## Materials and methods

### Protein expression and purification

Full length wild-type yARF1 (WT-yARF1) was cloned into a pET20(b) (Novagen, Inc) vector using NdeI and XhoI sites with a Hisx6 tag on the C-terminus. This construct was transformed into BL-21(DE3) competent cells (Stratagene, now part of Agilent Technologies) for expression. Cells were grown in M9 medium containing 1g/L ^15^NH_4_Cl, 2g/L glucose, and 100mg/L ampicillin at 37^°^C until OD_600nm_ reached 1.0~1.1 at which point isopropyl-β-D-thiogalactopyranoside (IPTG, 0.4mM) was added and cells were grown at 28^°^C overnight. Following cell lysis by French press and clarification by ultracentrifugation, proteins were purified by affinity chromatography on a HisTrap column and then by ion-exchange chromatography on a Q-Sepharose column. A final yield of 20~30mg of protein per liter of culture was typically achieved.

Full length yARF1 from a codon-optimized (CO-yARF1) gene (IDT DNA, Inc.) was cloned into pET20(b) using NdeI and XhoI sites with a C-terminal Hisx6 tag. Protein expression and purification followed the same protocol as for the wild-type yARF1 except that 1g/L ^14^NH_4_Cl was used in the M9 medium. The final yield was ~ 10mg per liter of culture.

### Quantification by mass spectrometry

^15^N (99%) WT-yARF1 and ^14^N CO-yARF1 were expressed and purified separately, then mixed at about a 1:1 concentration ratio based on OD_280nm_ absorption. The mixture was buffer-exchanged into 50mM NH_4_HCO_3_ by repeated dilution and concentration in a centrifugation concentration cell to a final volume of 0.1mL at ~2mg/mL (~1mg/mL for each protein). Following heat denaturation at 80^°^C for 1 hour, sequencing grade modified trypsin (Promega, Inc) (3μg) dissolved in 50mM acetic acid at 0.1μg/μL was added for overnight digestion at 37^°^C. The resulting peptide mixture was diluted to 20μM in 50% acetonitrile, 0.1% formic acid. The peptide mixture was analyzed by direct infusion using a Q-Tof 2 mass spectrometer (Waters, Milford MA) with a nanoelectrospray ionization (nESI) source. The peptide mixture was infused at a flow rate of 1μl/min, with a capillary potential of 3.8 kV and standard declustering potentials. MS Profile settings were set to 200, 900, and 1600 Th, with zero dwell time and 50% ramp time allocated for each transition to insure optimum quantitative response for peptide isotopomers while maintaining reasonable sensitivity for all peptides analyzed. Observed peaks were smoothed twice by the Savitzky Golay method, then the top 80% of each peak was centroided with the peak areas calculated for quantitation using the MassLynx software (Waters, Milford MA). For peptides where multiple charge states were detected, the charge state with the highest signal to noise ratio was used in each case for quantitation; the sole exception is peptide 83–96, where the ^13^C isotopic distribution and asymmetric peak shapes observed in the highest signal to noise ratio 1+ charge state of the unlabeled codon-optimized peptide strongly suggested an unresolved overlapping signal, prompting us to use the 2+ charge state for quantitation despite a slightly lower signal to noise ratio. Direct infusion spectra were divided into ten technical replicates, each replicate consisting of all scans obtained over one minute, and used for statistical analyses. The ratio of ^15^N-labeled, unsubstituted peptide signal intensity versus unlabeled, unsubstituted peptide signal intensity for all peptides not containing arginine were pooled for all technical replicates and used to determine the mean and variance for peptides that are not prone to substitution. Then, the ratios for arginine-containing peptides for each of the ten technical replicates were compared to the pooled arginine-free peptide ratios from the same sample, and an independent two-sample Student’s t-test was applied to determine two-tailed *p* values.

### Protein solubility test by SDS-PAGE

ARF mutants R72K and R78K were produced following the protocol described above. After cell lysis by French press, 50μL of cell lysate of each protein was pelleted by centrifugation at 16,000g in Eppendorf tubes. The supernatant was mixed with SDS-PAGE sample buffer at 10μL: 20μL ratio. The pellet was washed once with the French press buffer (25mM Tris (pH7.8), 1mM MgCl_2_, and 5mM β-mercaptoethanol) and re-clarified. The final pellet was dissolved in 50μL French press buffer plus 100μL SDS-PAGE sample buffer. After heating at 90^°^C for 3 mins, both supernatant and pellet samples were loaded at 10μL volume to a Tris-Glycine SDS-PAGE gel for electrophoresis. After gel staining with Coomassie blue, the intensity ratio of the yARF1 bands from the supernatant and pellet samples was interpreted as an indicator of protein solubility.

## Results

**Fig 2:**
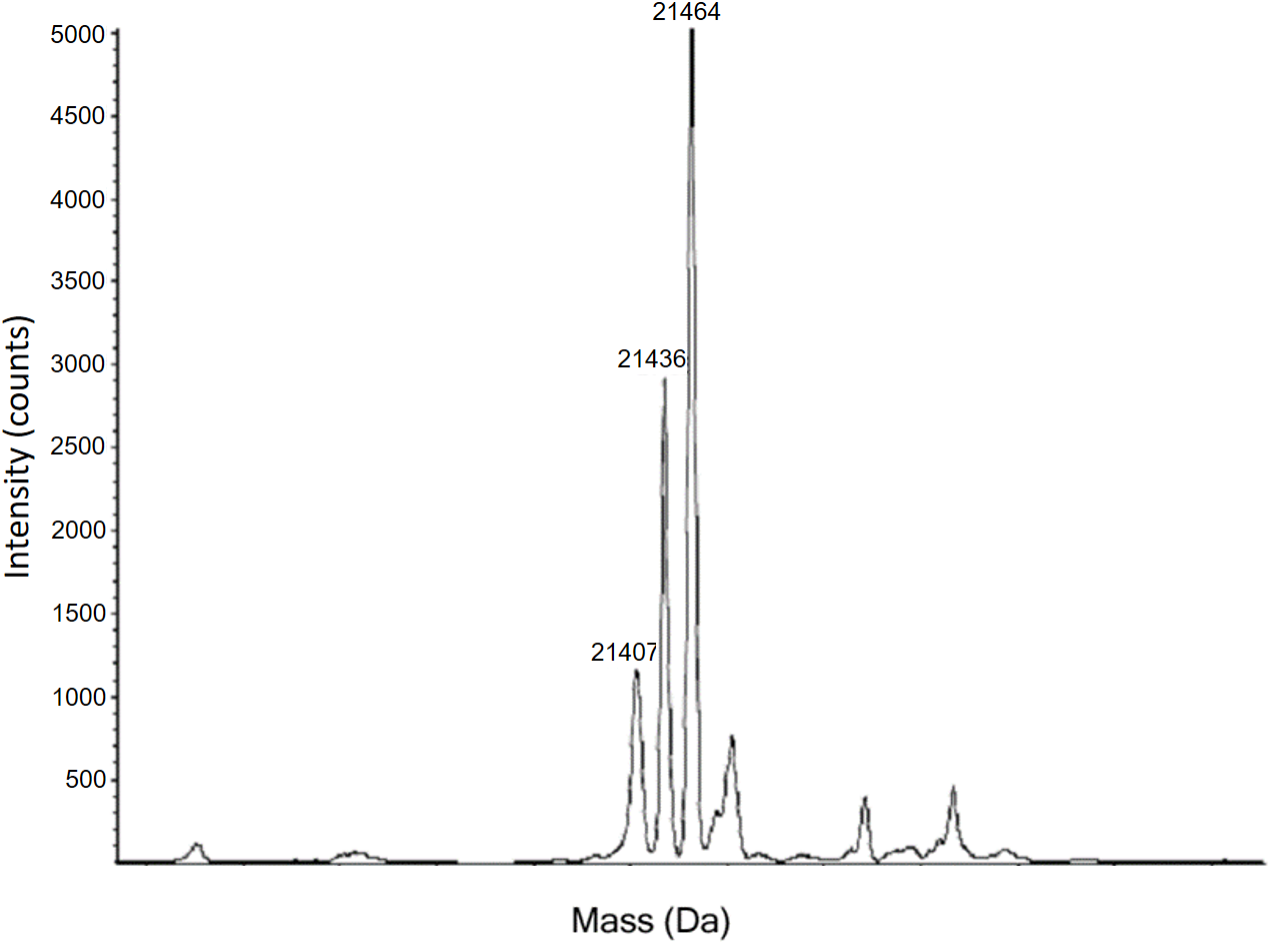
Mass spectrum of full-length yARF1. The non-substituted peak is at 21464 Da. The two substituted peaks are at 21436 Da (one substitution) and 21407 Da (two substitutions).

The first evidence of lysine substitution for arginine came from the mass spectrum of the intact protein expressed using the native (wild-type) sequence (see Fig 1). The electrospray ionization (ESI) spectrum of this product is presented in Fig 2. Wild-type yARF1 (expected mass 21463.4 Da) reveals 3 major peaks at 21407 Da, 21436 Da, and 21464 Da. The ~28Da mass difference corresponds to that between arginine and lysine. As WT yARF1 contains 9 arginine codons (AGA) that are rare in *E*. *coli*, a ladder pattern with separations of 28 Da is expected anytime lysine for arginine substitutions occur. Interestingly, fitting the 3 peaks in the WT yARF1 spectrum, based on a binomial probability mass function, failed to reproduce the experimental peak height ratios. Using a uniform 4.8% substitution probability, which matches the intensities of zero and one substitution peaks, the ratio of the one substitution to the two substitution peak is predicted to be 4.6. The observed ratio is 2.5, suggesting that the 9 arginine rare codon sites might have different levels of mis-incorporation. The 28 Da ladder pattern is qualitatively reproducible, but levels of substitution do seem to depend on growth conditions, with media in which amino acids are directly supplied showing lower levels of substitution. This phenomenon is worthy of further investigation. Also, because substituted and non-substituted proteins could have different ionization efficiencies in the above mass spectrometry analysis, the apparent sequence specific substitution in samples produced in minimal medium would benefit from a more quantitative analysis.

**Fig 3:**
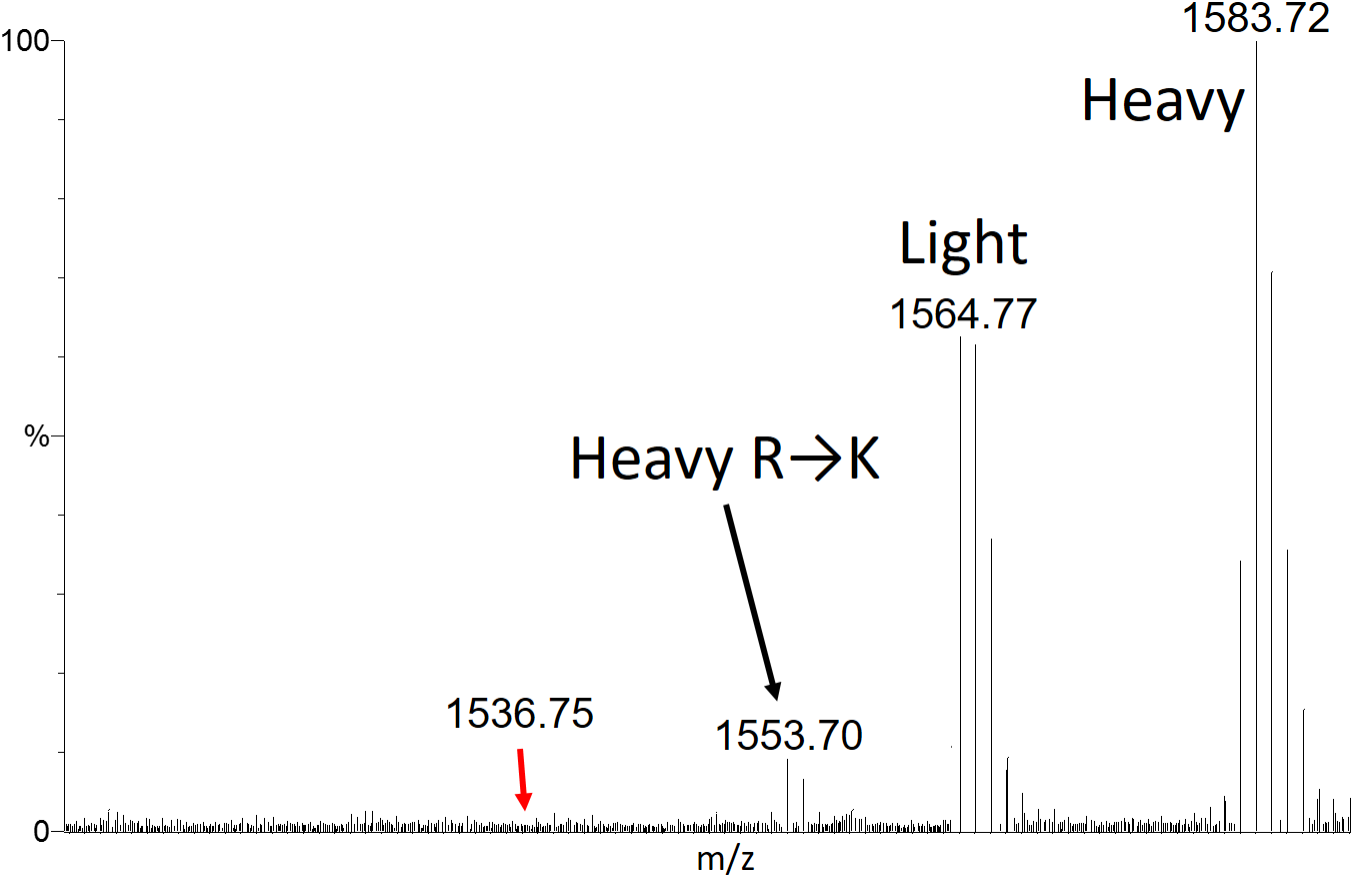
Mass spectrum of WT peptide 83–96. The peak from codon-optimized unlabeled yARF1 is labeled “Light”. The peak from unsubstituted ^15^N-labeled wild-type yARF1 is labeled “Heavy”, as is the peak from the R96→K substitution product. The m/z where the codon-optimized “Light” substitution product would appear (1536.75) shows no peak for any peptide measured.

**Fig 4:**
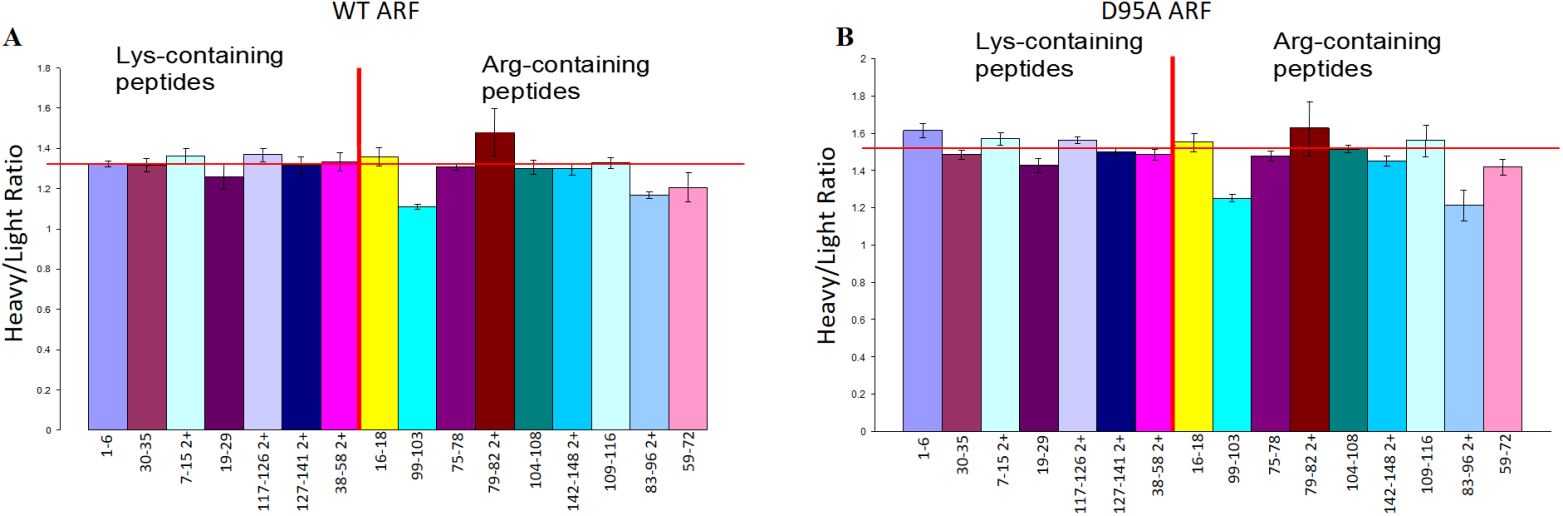
**Heavy to light ratios for (A) WT and (B) D95A yARF1.** Error bars represent two standard deviations from the mean. In each case, the charge state with the lowest local chemical noise level was chosen, except for peptide 83–96 where the ^13^C isotopic envelope is distorted because of spectral overlap. Also, in the WT 83–96 peptide, the Heavy peptide overlaps with the substituted Heavy 59–72 peptide, which may lead to under-estimation of substitution.

To gain quantitative insights into the site-specific substitution rates, we adopted an approach similar to the SILAC (Stable Isotope Labeling by Amino Acids in Cell Culture) approach used to quantify protein expression differences in cell culture experiments [31–33]. WT-yARF1 was expressed in medium containing ^15^NH_4_Cl (99%); thus, ^15^N labeled (heavy) proteins were produced. Codon-optimized yARF1 (CO-yARF1) was expressed in medium containing natural abundance NH_4_Cl; thus, ^14^N labeled (light) proteins were produced. The use of labeled ammonium chloride rather than a labeled amino acid provides large mass differences in peptides, with a wider choice in reference peptides, and it avoids possible overproduction of arginine carrying tRNAs under arginine supplementation conditions. The two proteins were purified separately and later mixed at roughly a 1:1 ratio. The mixture was then completely trypsin-digested and subjected to direct infusion nESI on a Q-Tof 2 mass spectrometer. For ^15^N WT-yARF1, two “heavy” peaks were observed for every arginine-containing peptide that had undergone significant substitution (Fig 3) with ~30 Da mass difference, corresponding to the non-substituted (heavy__ARG_) and the substituted (heavy__LYS_) residues. For ^14^N CO-yARF1, only one “light” peak was observed for each corresponding peptide due to the absence of substitution in the codon optimized gene. To eliminate variations due to ionization efficiency of the substituted and unsubstituted peptides, the peak area ratio of “heavy__ARG_” over “light__ARG_”, instead of “heavy__LYS_” over “heavy__ARG_”, was used to reflect substitution frequency. To compensate for the difference in total amounts of WT-yARF1 and CO-yARF1, the heavy/light ratios of non-substituted lysine containing peptides were used as the reference. As shown in Fig 4, the reference ratios are relatively constant around 1.32, indicating that WT-yARF1 is ~32% more abundant than CO-yARF1 in the mixture used in these analyses. In contrast, heavy__ARG_/light__ARG_ ratios for the 8 detected rare arginine codon containing peptides vary more significantly (Fig 4). Of these, peptide 59–72 (R72), 83–96 (R96), and 99–103 (R103) undergo significant substitution as suggested by p values less than 0.001 while 75–78 (R78), 79–82 (R82), 104–108 (R108), 109–116 (R116) and 142–148 (R148) have minimal levels of substitution. The rare codon containing peptide 73–74 was not observed. These values are summarized in Table 1.

**Table 1:** Substitution rate and significance of Arg→Lys substitutions. The degree of substitution in each peptide was determined as described under Materials and Methods. Statistical analysis used a two-sample Student’s t-test using ten independent replicates to determine two-tailed *p* values.

Site	Peptide	% Substitution	*p*
16–18	EMR	<1^†^	0.0089
59–72	NISFTVWDVGGQDR	8.086680761	<0.0001
75–78	SLWR	1.381757777	0.1645
79–82	HYYR	<1^†^	<0.0001^‡^
83–96	NTEGVIFVVDSNDR	12.02808819	<0.0001
99–103	IGEAR	16.17336152	<0.0001
104–108	ENMQR	1.827242525	0.0679
109–116	MLNEDELR	<1^†^	0.809
142–148	IGLHSIR	2.544548475	0.0126
D95A 83–96	NTEGVIFVVDSNAR	21.04779412	<0.0001

^†^The ratios of these peptides were higher than the mean.

^‡^This peptide had the lowest signal:noise ratio of all peptides measured (~3:1).

Several controls were run to eliminate trivial explanations for the variations and to test some of the more mechanistically based explanations, detailed below. First, it is possible that certain proteins with substitutions have different solubility or isolation properties. This could skew isotope ratios simply due to differences in the amount of protein isolated. For example, the disappearance of an arginine containing peptide could be due to insolubility of a protein with a lysine substitution at another site. Such difference is unlikely with the very conservative Lys for Arg substitution, but two mutant proteins with a deliberate substitution of Lys for Arg were made to test this possibility. R72 and R78, which show up as high and low frequency sites respectively, were both mutated to Lys. No difference in solubility could be detected, confirming that differential mis-incorporation does occur, at least between these two rare codon sites.

A second control was run on R96 which has a particularly high substitution rate. It is preceded by an aspartic acid, as is R72, another residue which has a moderately enhanced substitution rate. Aspartic acid is among the preceding amino acids slowing peptide bond formation to the largest extent in in vitro studies [27]. The aspartic acid was mutated to alanine (D95A) to remove this possible effect and a Mass Spec analysis on the extent of Lys for Arg substitution at R96 was performed. If any change resulted, the extent of substitution was increased (Fig 4, Table 1). The substitution levels of the other rare arginines were not appreciably affected by this mutation, supporting the reproducibility of sequence-specific mis-incorporation among different batches of protein.

## Discussion

The data presented show significantly enhanced lysine substitution at three of the nine arginine rare codon sites, R72, R96, and R103. There seems to be no trivial explanation for experimental variation due to properties of the substituted proteins or detectability of derived peptides. Therefore, the results must reflect, in some way, mechanistic aspects of the translation process. Following kinetic models such as those introduced by Hatzimanikatis [19, 20] or Lipowsky [18], and the specific kinetic factors suggested by work of the laboratories of Rodnina [24], Green [25] and Eherenberg [26], we can suggest several ways that levels of mis-incorporation at different sites could vary. This could be the result of a direct effect on binding free-energies or kinetic activation energies by ribosomal interactions with adjacent parts of the mRNA or nacent polypeptide, or it could be the result of sequence specific slowing of one of the steps at, or following, GTP hydrolysis, which would minimize the effect of this secondary selection step. Among possible sources for sequence specific slowing are: peptide bond formation by the preceding amino acid, limitations on the transit of the nacent peptide through the ribosomal tunnel, the slow progression of polysomal synthesis where a ribosome at a more C-terminal site stalls movement of ribosomes at more N-terminal sites, or the need to unravel mRNA secondary structure toward the 3’ end to allow progression of a ribosome down the sequence. The local concentration of the appropriate tRNAs could also be depleted in regions where a particular rare codon is clustered allowing inappropriate tRNAs to more effectively compete for an initial binding site. We can eliminate some of these possibilities based on the experiments conducted and examination of the yARF1 sequence.

In yARF1 R72 and R96 sites are high substitution frequency targets and both are preceded by aspartic acids. The respective codons used for the aspartic acids are GAC and GAT. The fact that they are different argues for a direct role for the amino acid as opposed to the codons. The carboxylate of the aspartic acid side chain is believed to exert an electrostatic effect making the carboxyl carbon less electrophilic and slowing nucleophilic attack by the incoming amine [27]. This could stall addition of amino acids at sites immediately following aspartates and promote inappropriate incorporation by eliminating the effects of more rapid processing of cognate tRNAs. To test this hypothesis, the aspartic acid codon in front of R96 was replaced with an alanine codon, and if anything, enhanced rather than diminished errors were observed (Fig 4, Table 1). Thus, this mechanism is not likely the cause for increased mis-incorporation rates at R72 and R96. However, there may be other sequence related effects. For example, most of the sites with low substitution frequency have bulky hydrophobic preceding residues, such as isoleucine (R148), tryptophan (R78), leucine (R116), and tyrosine (R82). This may directly affect differential interactions of cognate and near cognate tRNAs with ribosomal machinery to lower mis-incorporation rates.

With respect to the possibility of a polysomal stalling, examination of the mRNA sequence does show a tight cluster of rare codon sites (four arginines and one leucine from 72 to 82) and a less tight cluster from 96 to 116. If stalling of N-terminal ribosomes in a polysome cluster occurred in these regions, we would expect fewer errors well away from the cluster and more errors near the beginning of the cluster. R18 is, in fact, one of our least error prone sites, but it is not coded by AGA and would suffer less from inherent cognate tRNA deficiencies in *E*. *coli* in any event. R148 is fairly far away from these regions and has a relatively low rate of miss-incorporation. R72 is at the beginning of the tight cluster, and we do see a significant increase in miss-incorporation. R96 and R103 have high miss-incorporation rates and are at the beginning of the less tight cluster. Thus there is some support for an effect of polysomal stalling.

A possible correlation with predicted mRNA secondary structure can likewise be examined. Formation of a downstream stem-loop or pseudoknot structure of the mRNA during the translation process could stall ribosome movement and contribute to variation in translation rates as a function of position [34]. If these structures are formed during translation they could retard steps subsequent to GTP hydroylsis and accentuate incorporation of inappropriate amino acids. Based on the crystal structure of the ribosome/mRNA complex [35], the A site triplet is located at +4 to +6 and the mRNA entrance position is at +13 to +15. If the sequence next to the entrance site (*e*.*g*., +16 to +18) is involved in any secondary structure formation, a longer pause will be expected at the A site. Furthermore, according to Wen *et al*, the ribosome opens up exactly 3 base pairs (*i*.*e*., +16 to +18 plus certain complementary sequence further down the mRNA) prior to translocation. The participation of the +16 to +18 sequence in secondary structure formation could prolong the pause before movement while secondary structure immediately after +16 to +18 is not expected to have a significant effect. In yARF1, the 8 detected rare arginines occur at mRNA position 346–348^*^(R72), 364-366(R78), 376-378(R82), 418–420^*^(R96), 439–441^*^(R103), 454-456(R108), 478-480(R116), and 574-576(R148) (high frequency sites are noted by ^*^). The pauses at these sites should be correlated with the secondary structure forming potential at, respectively, 358–360, 376–378, 388–390, 430–432, 451–453, 466–468, 490–492, and 586–588. Of these, 358–360 can anneal with 443–438 (anti-parallel), 451–452 (2 out of 3 bases) with 607–606, 490–492 with 596–594, and 586–588 with 529–527, based on a RNA secondary structure prediction from the GeneBee server. The former two sites correspond to high frequency sites while the latter two to lower frequency sites. Hence no strong correlation is found between substitution and secondary structure by this analysis.

Local depletion of arginine charged tRNAs also provides a possible explanation for our observations. It is known that individual translation steps can be quite fast (10–15 aas per second) and translation by polysomes (multiple ribosomes on a single mRNA) should also contribute to the potential for local depletion of tRNAs. Again, examination of the sequence shows a cluster of four arginine sites from amino acids 72 to 82 and another less tight cluster of four from 96 to 116. It is hard to predict where in these clusters depletion effects would be observed. However, the three most error prone sites do occur at the beginning of each of these regions. While we can’t exclude the possible effects of preceding hydrophobic amino acids mentioned above, or more general interactions of the ribosome with extended parts of mRNA or nascent peptide, we believe that polysome effects, whether from codon depletion or ribosomal stalling in clustered regions of rare codons, provide a probable explanation for our observations.

In any event, the simple observation of differential incorporation of inappropriate amino acids within a set of nine identical rare codons in the eukaryotic protein, yARF1, provides some fascinating data that can reflect on the detailed mechanism of mRNA translation and improve modeling of this process. Given the small sample requirements of mass spectrometry, it may be feasible to do a systematic study of mRNA with codon variation upstream and downstream of rare codons. This would clearly contribute to an improved ability to discriminate mechanisms. The methods presented can also potentially be extended to a quantitative assessment of mis-incorporation rates at other rare codon sites, and the effects of these sites in hosts other than E. coli. There are also practical implications, including improving the efficiency of heterologous expression of biologicals in the pharmaceutical industry and minimizing mis-incorporation of amino acids in the products. Quite aside from mechanistic studies, it is important to remind the structural biology community not only of the occurrence, but also the magnitude, of translational mistakes observed in expression of heterologous proteins in E. coli. These mistakes can lead to misinterpretation of minor signals in NMR spectra and they can interfere with crystallization or degrade resolution in X-ray studies. Hopefully the studies and discussion presented here will serve this purpose as well.

It is important to remember that the data are being acquired under the stress of heterologous expression and that the codons examined would not be rare in a native host. While the magnitudes of the effects will be reduced under native conditions, we believe fundamental mechanisms will still be operative and the insight useful. There are also rare native codons in many proteins. In yARF1 there are two natively rare arginine codons, those for R18 and R98 that use the CGU as opposed to AGA, (see Fig 1). These could be present to accommodate an important intermediate folding step or perhaps promote the essential addition of the N-myristoyl modification that would normally occur in homologous expression of yARF1. It is interesting that our codon-optimized version of yARF1, while minimizing mistakes in translation, did not actually improve expression in bacteria. The fact that these natural rare codons are not rare in *E*. *coli*, and an essential slowing of translation may have been removed, could potentially be the cause.
